# Infusion of two-dose mesenchymal stem cells is more effective than a single dose in a dilated cardiomyopathy rat model by upregulating indoleamine 2,3-dioxygenase expression

**DOI:** 10.1186/s13287-022-03101-w

**Published:** 2022-08-12

**Authors:** Chenyi Gong, Lei Chang, Xuan Sun, Yu Qi, Rong Huang, Ke Chen, Bin Wang, Lina Kang, Lian Wang, Biao Xu

**Affiliations:** 1 Department of Cardiology, Nanjing Drum Tower Hospital, State Key Laboratory of Pharmaceutical Biotechnology, Medical School of Nanjing University, No. 321 Zhongshan Road, Nanjing, 210008 China; 2 Department of Cardiology, Nanjing Drum Tower Hospital, Clinical College of Nanjing Medical University, Nanjing, 210008 Jiangsu China; 3 Clinical Stem Cell Centor, Nanjing Drum Tower Hospital, Medical School of Nanjing University, No. 321 Zhongshan Road, Nanjing, 210008 China

**Keywords:** Human umbilical cord mesenchymal stem cells, Rat dilated cardiomyopathy, Myocardial inflammation, Indoleamine 2,3-dioxygenase, Regulatory T cells

## Abstract

**Background and aims:**

The therapeutic efficacy of single-dose mesenchymal stromal cell (MSC) therapy for heart failure (HF) remains inconsistent. This study aimed to investigate whether infusion with two-dose human umbilical cord MSC (hUCMSCs) could be therapeutically superior to single-dose therapy in a rat model of dilated cardiomyopathy (DCM) and explored the underlying mechanisms.

**Methods:**

Male Sprague–Dawley rats were intraperitoneally injected with doxorubicin (DOX) to establish a DCM model and randomized to intravenously receive single-dose or two-dose hUCMSCs at an interval of 14 days. Their left ventricular (LV) systolic and diastolic functions were analyzed by echocardiography. The percentages of Th1, Th2, Th17, and Treg cells in the heart, spleen, lymph nodes, and peripheral blood and the levels of serum cytokines in individual rats were analyzed by flow cytometry and cytometric bead assay, respectively. The degrees of cardiac fibrosis and cardiomyocyte apoptosis were examined by histology. The importance of indoleamine 2,3-dioxygenase (IDO), an activator of Treg differentiation, in the therapeutic effect of hUCMSCs on inflammation and heart function of rats was determined after induction of IDO over-expression (IDO-OE) using IFN-γ (1 ng/ml) and TNF-α (10 ng/ml) stimulation or silencing (IDO-KD) using small interfering RNA (siRNA) technology.

**Results:**

Compared with the single dose, two-dose hUCMSCs were more effective in improving LV performance, attenuating cardiac dilation, reducing cardiomyocyte apoptosis and cardiac fibrosis. Two-dose hUCMSC therapy significantly increased Treg number in the heart and peripheral blood, accompanied by increased cardiac IDO expression. Compared with the control hUCMSCs, IDO-OE hUCMSCs significantly enhanced Treg and Th2 cell responses and decreased systemic Th17 cell responses and Th1 cell numbers in the mediastinal lymph nodes. Treatment with IDO-OE hUCMSCs significantly improved LV remodeling and dysfunction. However, treatment with IDO-KD hUCMSCs had opposite effects in rats.

**Conclusions:**

Administration of two-dose hUCMSCs has better therapeutic effects than single-dose therapy for inhibiting myocardial inflammation to improve LV function in DCM rats. These effects are associated with upregulating IDO expression and its systemic anti-inflammatory activities.

**Supplementary Information:**

The online version contains supplementary material available at 10.1186/s13287-022-03101-w.

## Introduction

Inflammation is crucial for the pathogenesis of chronic heart failure (HF) [1]. Low-grade chronic inflammation contributes to the pathogenic progression of established chronic HF in human patients [2], while emerging elevated inflammatory biomarkers become a hallmark of chronic HF [3, 4].

Due to the limited self-regenerative capacity of the heart, several preclinical studies have attempted to explore the potential mesenchymal stromal cell (MSC)-based therapies for promoting myocardial self-repair and restoring cardiac function in the injured heart of rodents [5–10], as MSCs are easy to be isolated from multiple sources and have low immunogenicity, minimum ethical concerns, and greater proliferative capacity [11]. However, the therapeutic effects of MSCs on HF in human randomized clinical trials remain controversial or inconsistent [4]. Considering its technical difficulty and related risk of intracoronary or intramyocardial injection with MSCs, most clinical trials apply intravenous injection with single-dose MSCs, which might lead to poor engraftment of MSCs into the heart, as most of the infused MSCs usually accumulate in the lung and die soon after transplantation [12]. Currently, there is still limited information on the therapeutic efficacy of repeated dosing of MSCs on HF, although the intravenous route of repeated dosing of MSCs is possible and safe.

MSCs have been demonstrated to have immunosuppressive capability that is licensed by pro-inflammatory interferon-γ (IFN-γ) and tumor necrosis factor-α (TNF-α) and dependent on upregulated indoleamine 2,3-dioxygenase (IDO) expression and activity [13]. The IDO and its metabolite kynurenic acid are activators of Treg differentiation [14–16]. Tregs can migrate and be resident in the heart to promote functional repair and to protect from myocardial infarction and ischemic injury in humans and rodents [17–19]. However, it is still unclear whether infusion with two-dose human umbilical cord MSCs (hUCMSCs) is more effective than one dose in improving cardiac function and whether this benefit depends on the expression of IDO and its modulation on inflammation.

Given that repeated intravenous administrations of MSCs are feasible and can be routinely applied in clinical practice, this study aimed to compare the effects of two-dose with one dose hUCMSCs on inflammatory responses in a rat model of dilated cardiomyopathy (DCM) induced by doxorubicin (DOX). The results revealed that intravenous treatment with two-dose hUCMSCs led to a better therapeutic efficacy in promoting functional repair and mitigating inflammation in rat hearts by upregulating IDO expression. These findings may be valuable for clinical translation.

## Materials and methods

All procedures involving animals were approved by the Institutional Ethics Committee of Nanjing Drum Tower Hospital (Approval No. 20210116) and performed in accordance with the guidelines of the Guide for the Care and Use of Laboratory Animals published by the National Institutes of Health (Eighth Edition).

### Animals

Eight-week-old male Sprague–Dawley (SD) rats at 200 ± 20 g were purchased from the Model Animal Research Center of Nanjing University and maintained in a controlled environment at ∼23 °C with 40–50% humidity under a 12/12 h dark/light cycle.

### Isolation and identification of hUCMSCs

All umbilical cord (UC) samples were taken after their parents signed the written informed consent, and the study was approved by the Research Ethics Board of Nanjing Drum Tower Hospital (permit number: 2017–161-01).

Briefly, a 15-cm-long fresh UC was obtained from a full-term delivery donor, stored in sterile PBS supplemented with 100 IU/mL of penicillin and 100 μg/mL of streptomycin (PS, Gibco, USA), and immediately transferred to the GMP cell facility in a shipment box at 2–6 °C. All samples were negative for hepatitis B virus (HBV), hepatitis C virus (HCV), human immunodeficiency viruses (HIV I and II), syphilis, cytomegalovirus (CMV), and Epstein–Barr virus (EBV).

MSCs were isolated from individual UC samples and cultured as described previously [20]. Briefly, MSCs were cultured into cell culture flasks in low-glucose DMEM supplemented with 10% fetal bovine serum (FBS, complete medium) in a 5% CO_2_ incubator at 37 °C. The medium was changed every two days. At 80–90% confluency, the monolayer of cells was digested with TrypLE (Gibco, USA) and harvested as the first passage. The cells at passages 3–5 were used for subsequent experiments. The hUCMSCs were identified by flow cytometry using specific cell surface markers, including CD105(+), CD73(+), CD90(+), CD11b(−), CD34(−), CD45(−), CD19(−), and HLA-DR(−).

### Modulation of IDO expression in hUCMSCs

The hUCMSCs were treated with IFN-γ (1 ng/ml) and TNF-α (10 ng/ml) for 24 h to induce IDO over-expression [13]. The control hUCMSCs were treated with vehicle. Alternatively, hUCMSCs were transfected with control scramble siRNA or IDO-specific siRNA (5′-GAACGGGACACUUUGCUAAdTdT-3′, RiboBio, Guangzhou, China) using Lipofectamine 2000, according to the manufacturer’s instruction (11668-019; Invitrogen, Carlsbad, CA, USA). Briefly, hUCMSCs in the logarithmic phase of growth were trypsinized, harvested, and cultured into a 6-well plate overnight. At ~ 80% confluence, the cells were transfected with each type of siRNA at 50 nM and cultured for 24 h. Other control hUCMSCs were treated with vehicle.

### A DCM rat model and hUCMSCs administration

**Fig. 1:**
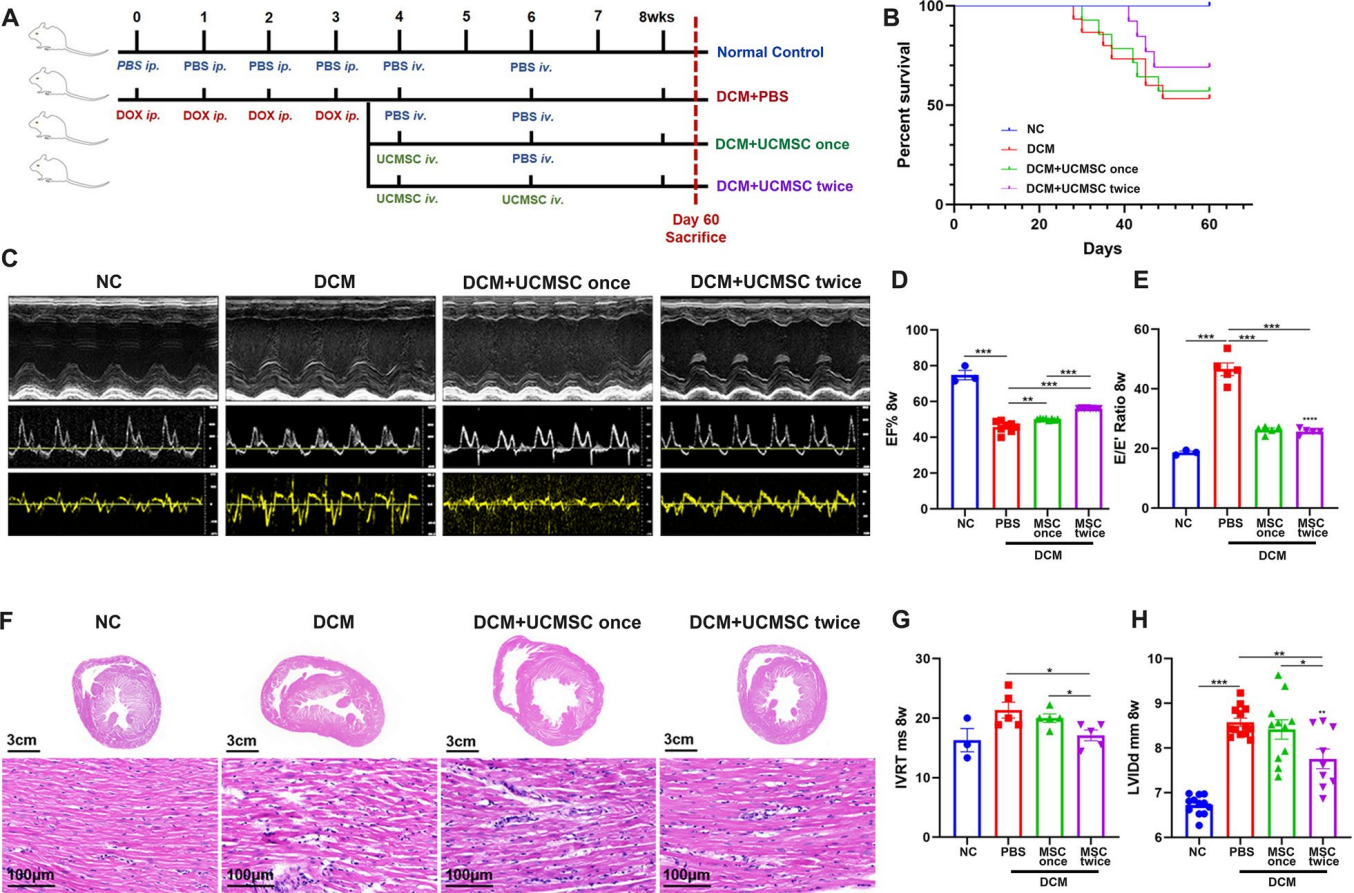
Two-dose intravenous administration of hUCMSCs improves cardiac function in DCM rats. **A** A diagram illustrated the experimental design. **B** Kaplan–Meier survival curves of four groups of rats. **C** Representative images of M-mode echocardiography. **D**, **E**, **G**, **H** Echocardiography analysis of diastolic function (IVRT, E/E′), systolic function (EF%), and left ventricular internal diameter at end diastole (LVIDd). **F** Representative images of hematoxylin and eosin (H&E) staining in rat hearts. H&E, the upper images scale bar = 3 cm, the bottom images, scale bar = 100 μm. Data are represented as mean ± SEM of each group (*N* = 3–10 rats per group) from at least two separate experiments. *P < 0.05, **P < 0.001, ***P < 0.001 by one-way ANOVA

To induce DCM, SD rats were injected intraperitoneally with 1.75 mg/kg doxorubicin (DOX, Sigma, St. Louis, USA) in saline for four times with an interval of seven days [21]. Subsequently, the rats were randomized and administered intravenously with control PBS, single dose of hUCMSCs (2 × 10^6^ in 500 μl PBS) at 4 weeks post-beginning induction or two-dose hUCMSCs at 4 and 6 weeks post-induction into the tail vein (Fig. 1A) [22]. All rats were euthanized at 60 days post-induction.

### Echocardiography

Individual rats were sedated with 2–2.5% isoflurane, and their cardiac function was evaluated by 2-dimensional transthoracic echocardiography using a Visual Sonic Ultrasound system with a 30-MHz transducer. The heart was imaged in the parasternal short-axis view at the level of papillary muscles to record M-mode measurements and to determine heart rate, wall thickness, and end-diastolic and end-systolic dimensions. The left ventricular ejection fraction (EF%) and left ventricular fractional shortening (FS%) were calculated as indexes of cardiac contractile function. The diastolic function of rats was assessed by trans-mitral flow Doppler from the apical 4-chamber view to measure the E/A ratio, isovolumic relaxation time (IVRT), and E/E′ ratio.

### Immune cell isolation and flow cytometry

Live mononuclear cells were isolated from the peripheral blood, heart, spleen, and lymph nodes (LNs) for flow cytometry, as previously described [23]. Briefly, the collected hearts were digested with collagenase and filtered through 40 μm filters to remove extracellular connective tissue debris, followed by centrifugation. The spleen and LNs were finely minced using a scalpel and filtered through 100 μm filters. The cells were fixed with 1% paraformaldehyde and stained with FITC-anti-CD4 for 1 h on ice. The cells were permeabilized with 0.5% Tween-20 for 20 min and intracellularly stained with PE-anti-CD25, APC-anti-Foxp3, APC-anti-IFNγ, PE-anti-IL-4, and PE-Cy7-anti-IL-17 for 45–60 min on ice. Finally, the cells were stained with the Fixable Viability Dye eFluor 506 (eBioscience) to exclude dead cells. After being washed, the cells were analyzed by flow cytometry using a BD FACS Aria II Flow Cytometer. The data were analyzed using FlowJo software v10.0.6.

### Western blot

Rat hearts were homogenized in RIPA buffer supplemented with protease inhibitors and centrifuged at 15,000 g for 10 min at 4 °C. After determination of protein concentrations by the BCA method, individual heart lysates (30 µg/lane) were separated by sodium dodecyl-sulfate polyacrylamide gel electrophoresis (SDS-PAGE) on 10% gels and transferred onto a polyvinylidene difluoride (PVDF) membrane. The membranes were blocked with 5% BSA in TBST for 2 h at room temperature and incubated overnight at 4 °C with primary antibodies. The primary antibodies were against cleaved caspase-3, caspase 3, Bax, Bcl-2, MMP9, α-SMA, and GAPDH at 1:1000 dilution (Cell Signaling Technology). After being washed, the bound antibodies were detected with horseradish peroxidase (HRP)-conjugated secondary antibodies at room temperature for 2 h and visualized with the ECL reagent. The relative levels of protein expression to the control GAPDH were quantified by densitometric scanning using ImageJ software.

### Gene expression analysis by real-time quantitative PCR (RT-qPCR)

Total RNA was extracted from rat hearts, spleens, or LNs using Total RNA Extraction Reagent (Vazyme Biotech, Nanjing, China) and reverse-transcribed into cDNA using the HiScript III RT SuperMix for qPCR (Vazyme Biotech, Nanjing, China). The relative levels of interesting gene to the control GAPDH mRNA transcripts were quantitated by RT-qPCR in an Applied Biosystems using ChamQ Universal SYBR qPCR Master Mix (Vazyme Biotech, Nanjing, China) and specific primers (Table 1). The obtained experimental data were analyzed by the 2^−ΔΔCt^ method.

**Table 1:** Real-time RT-PCR Primers

Gene	Forward primer	Reverse primer
Human		
*Gapdh*	TCAAGAAGGTGGTGAAGCAGG	TCAAAGGTGGAGGAGTGGGT
*Ido1*	GCCCTTCAAGTGTTTCACCAA	CCAGCCAGACAAATATATGCGA
Rat		
*Gapdh*	TGTGAACGGATTTGGCCGTA	ATGAAGGGGTCGTTGATGGC
*Ido1*	AGCACTGGAGAAGGCACTG	CGTGGAAAAAGGTGTCTGG
*Nppb*	CAATCCACGATGCAGAAGCTG	GGCGCTGTCTTGAGACCTAA
*Nppa*	TGGGTCTTGTTAGGGCTCAAACCT	TGAAACTCAAGGGACACCCATCGT
*Mmp2*	ACAACAGCTGTACCACCGAG	GGACATAGCAGTCTCTGGGC
*Mmp9*	GATCCCCAGAGCGTTACTCG	GTTGTGGAAACTCACACGCC
*Acta2*	CGCCATCAGGAACCTCGAGAAG	ATCATCACCAGCAAAGCCCG
*Col1a1*	GACTGTCCCAACCCCCAAAA	CTTGGGTCCCTCGACTCCTA
*Col3a1*	TCCCCTGGAATCTGTGAATC	TGAGTCGAATTGGGGAGAAT
*Timp1*	CAGCAAAAGGCCTTCGTAAA	TGGCTGAACAGGGAAACACT
*Tbet*	AACCAGTATCCTGTTCCCAGC	TGTCGCCACTGGAAGGATAG
*Gata3*	AGCCAGGAGAGCAGGGACG	CTGTTAATATTGTGAAGCTTGTAGTAGAG
*Rorγt*	CCCATCTATGAGGGTTACGC	TTTAATGTCACGCACGATTTC
*Foxp3*	ACCGTATCTCCTGAGTTCCAT	GTCCAGCTTGACCACAGTTTAT
*Ifng*	TACACGCCGCGTCTTGGT	GAGTGTGCCTTGGCAGTAACAG
*Tnfa*	AACTCGAGTGACAAGCCCGTAG	GTACCACCAGTTGGTTGTCTTTG
*Il2*	GATCCAGCGATACGAGAACTT	CTCTGCATCTGGACCGTCA
*Il4*	AGCGGATCTCGGAAACTCAAT	CTGGCAGTCTGACATC
*Il5*	GAGGGGGCACTGTGGAAATA	ACTCATCACGCCAAGGAACT
*Il6*	GCCTATTGAAAATCTGCTCTGG	GGAAGTTGGGGTAGGAAGGA
*Il10*	GAAGATCTCAATAGCGTCA	AATCTCTCAATCCTCGTC
*Il12rβ2*	TTGCATCGCTATCATCGTGG	CCTCTTTTGAAGCAATAGGG
*Il13*	CCTGGAATCCCTGACCAACA	GCCATAGCGGAAAAGTTGCT
*Il17*	GGTACTCATCCCTCAAAGTTCA	CTCTTCAGGACCAGGATCTCT
*Il33*	GTGCAGGAAAGGAAGACTCG	TGGCCTCACCATAAGAAAGG
*Ccr5*	CACCCTGTTTCGCTGTAGGAATG	GCAGTGTGTCATCCCAAGAGTCTC
*Tgfb*	CTGCTGACCCCCACTGATAC	AGCCCTGTATTCCGTCTCCT
*cTnI*	AAAAAGTCTAAGATCTCCGCCTCCA	GGTTTTCCTTCTCAATGTCCTCCTT
*cTnT*	CGTAGAAGAGGTTGGTCCTGATGAA	TGTACCCTCCAAA

### Histological analysis

The collected heart tissues were fixed in 4% formalin overnight and paraffin-embedded. The paraffin-embedded heart sections (4–5 μm) were deparaffinized, and rehydrated, followed by routine hematoxylin and eosin (H&E) staining and Sirius Red staining to evaluate the cardiac dilation and tissue fibrosis, respectively. Furthermore, the heart sections were subjected to antigen retrieval and stained with anti-CD3 and anti-CD68 to evaluate the contents of infiltrated T cells and macrophages. The frequency of apoptotic cells in heart tissue sections was quantified by terminal deoxynucleotidyl transferase-mediated deoxyuridine triphosphate nick-end labeling (TUNEL) assay using the TUNEL BrightGreen Apoptosis Detection kit (Vazyme Biotech, Nanjing, China), following the manufacturer’s protocol. Apoptotic nuclei were labeled with red fluorescein, and total nuclei were stained with DAPI. To visualize cardiomyocytes, the heart tissue sections were co-stained with FITC-anti-TnT (green) and examined under a confocal microscopy (LSM700, Zeiss, Jena, Germany). The percentages of apoptotic cells were calculated.

### Statistical analysis

Data from at least five independent experiments are presented as mean ± SEM, unless otherwise indicated. The difference among groups at a single time point was analyzed by two-tailed unpaired Student’s *t* test or one-way analysis of variance (ANOVA) and post hoc Tukey’s multiple comparisons test. The differences among groups at multiple time points were analyzed by two-way ANOVA and post hoc Bonferroni’s multiple comparison test. All analyses were performed using the Prism 8 software (GraphPad), and a *P* value of less than 0.05 was considered statistically significant.

## Results

### Two-dose intravenous administration of hUCMSCs improves cardiac function in DCM rats

Compared with the DCM control group, intravenous treatment with single-dose hUCMSCs failed to change 60-day mortality rate, but treatment with two-dose hUCMSCs tended to reduce 60-day mortality rate (30.77% vs. 42.86% in the DCM group) (Fig. 1B). Next, we examined the impact of hUCMSC treatment on DOX-related cardiotoxicity in rats by echocardiography. The results indicated overt LV systolic and diastolic dysfunctions, including reduced EF% (45.70%) and FS% (24.09%) and elevated IVRT (21.33 ms) and E/E′ (46.56) in the DCM group on day 56 post-induction, while treatment with single- or two-dose hUCMSCs improved LV function (Fig. 1C–E, G, H). Furthermore, the therapeutic effects of two-dose hUCMSCs on enhancing EF%, IVRT, and attenuating cardiac dilation were obviously better than single-dose hUCMSCs in rats (Fig. 1F). Therefore, two-dose intravenous administration with hUCMSCs improved cardiac function in DCM rats.

### Treatment with hUCMSCs increases treg responses and IDO expression in the heart

**Fig. 2:**
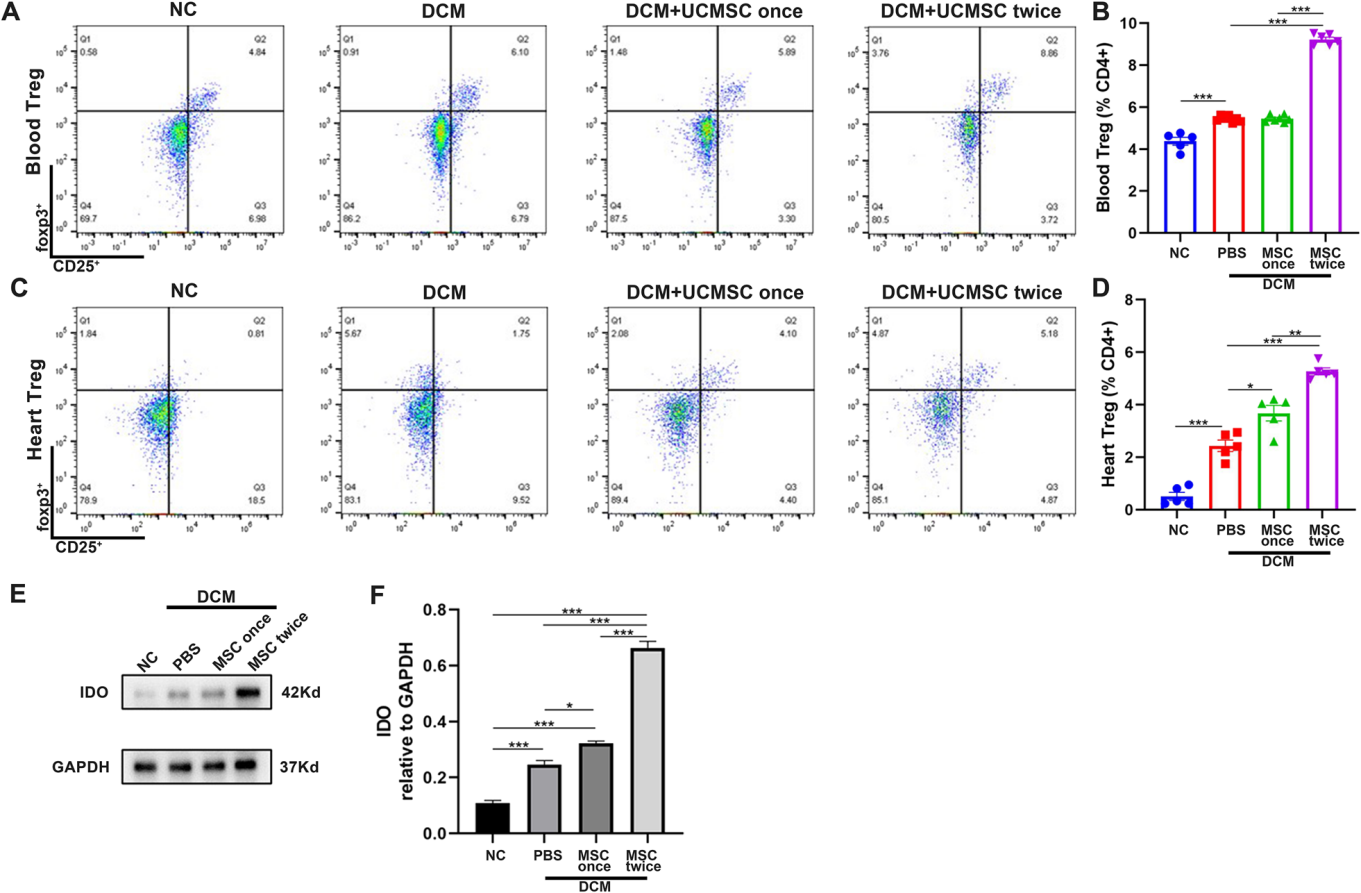
Two-dose intravenous administration of hUCMSCs increases the frequency of circulating and heart Tregs and IDO expression in the hearts of DCM rats. **A**, **B** Representative flow cytometry dot plots of CD4^+^CD25^+^foxp3^+^ Tregs in peripheral blood and quantitative analysis. **C**, **D** Representative flow cytometry dot plots and quantitative analysis of Tregs in the hearts of DCM rats at 8 weeks post-induction. **E**, **F** Western blot and quantitative analysis of IDO expression. Data are presented as mean ± SEM of each group (*N* = 3–5 rats per group) from at least two independent experiments. *P < 0.05, **P < 0.001, ***P < 0.001 by one-way ANOVA

Tregs can negatively regulate inflammation that is essential in the progression of many cardiovascular diseases [24]. Numerous studies have shown that down-regulated Treg infiltrates and impaired function of Tregs are associated with the development of HF [25–27]. Accordingly, we analyzed the proportions of CD4 + Foxp3 + Tregs in peripheral blood and the heart of rats at 8 weeks post-induction by flow cytometry. We found that hUCMSC therapy significantly increased the percentages of Tregs in both types of samples and the effects of two-dose hUCMSC therapy were significantly stronger than that of single-dose therapy in rats (Fig. 2A–D). As IDO enhances the differentiation of Tregs [14, 15, 28], we analyzed IDO expression in the heart of rats by Western blot. The results revealed that the levels of IDO expression in the hearts of rats, particularly in the two-dose hUCMSC group, significantly increased, relative to that in the DCM group of rats (Fig. 2E, F). These data indicated that two-dose hUCMSC treatment significantly upregulated IDO expression and increased the percentages of Treg infiltrates in the heart and circulation of DCM rats, which may alleviate systemic inflammation.

### Treatment with hUCMSCs attenuates cardiac inflammation and modulates inflammation in rats, dependent on the levels of IDO expression in hUCMSCs

**Fig. 3:**
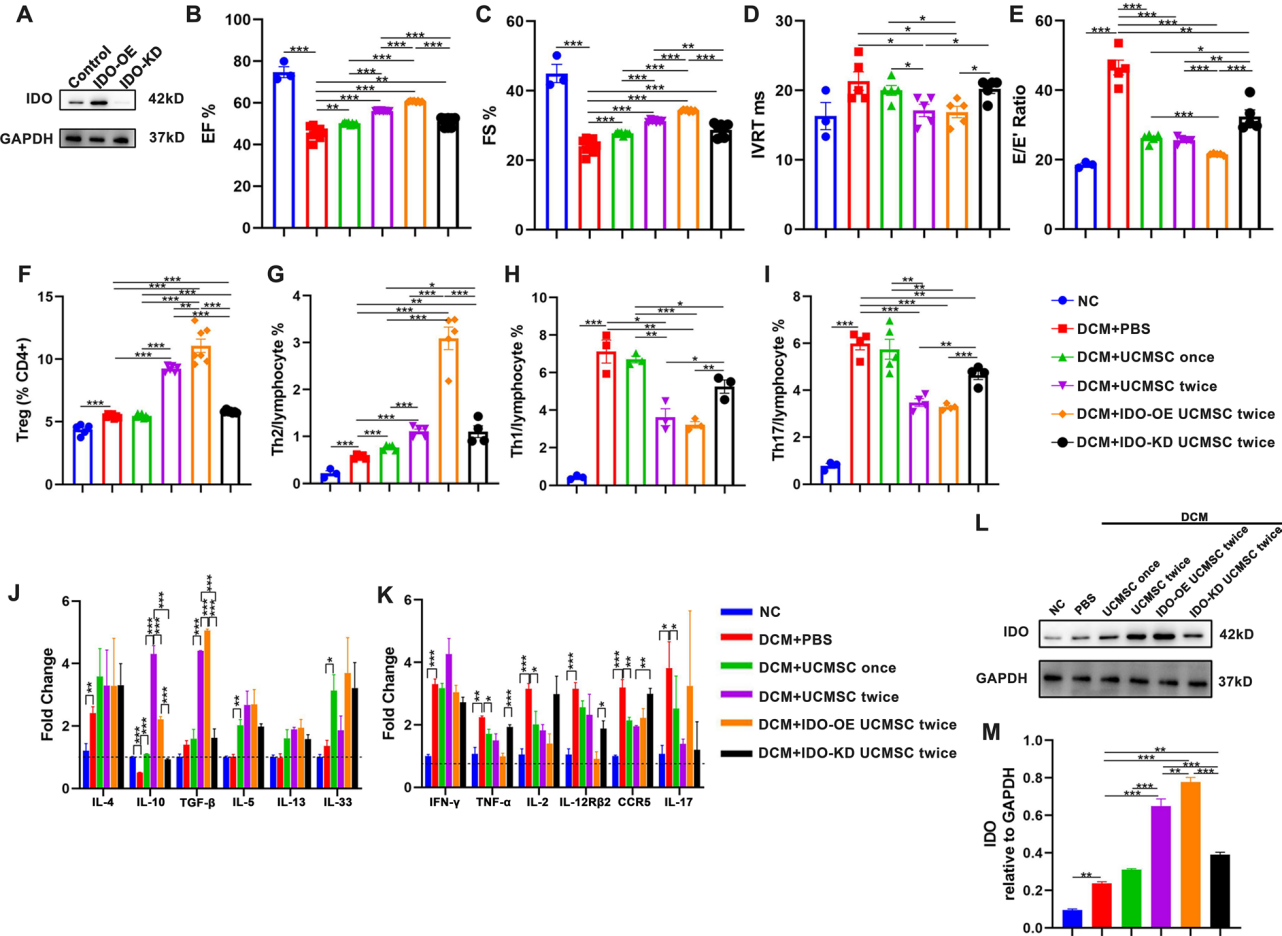
IDO-OE hUCMSC treatment attenuates cardiac inflammation and cardiac dysfunction in DCM rats. **A** Western blot analysis of IDO expression in the different groups of hUCMSCs. **B**–**E** Echocardiography analysis of both systolic (EF%, FS%) and diastolic (E/E′, IVRT) functions in DCM rats. **F**–**I** Flow cytometry analyses of the proportions of Tregs, Th2, Th1, and Th17 cells in the hearts of rats. **J**, **K** RT-qPCR analyses of the related mRNA transcript levels of the genes involved in Treg, Th2, Th1, and Th17 cell functions in the hearts of rats. **L**, **M** Cardiac IDO expression was assessed by Western blot and quantitatively analyzed. Data are mean ± SEM of each group (*N* = 3–5 rats per group) from at least three independent experiments. *P < 0.05, **P < 0.001, ***P < 0.001 by one-way ANOVA

To determine the importance of IDO expression in the therapeutic effect of hUCMSCs in DCM rats, we treated hUCMSCs with IFN-γ and TNF-α to induce IDO over-expression (DO-OE) or transfected hUCMSCs with IDO-specific siRNA to knockdown IDO expression (IDO-KD) (Fig. 3A). Compared with other group of cells, treatment with IDO-OE hUCMSCs obviously increased systolic and diastolic functions in DCM rats (Fig. 3B–E, Additional file 1: Fig. S1A, B). Flow cytometry revealed that compared with the NC group, the DCM group of rats displayed significantly higher frequency of cardiac Th1, Th2, Th17 cells, and Tregs, while treatment with hUCMSCs further increased the percentages of cardiac Th2 cells and Tregs, but decreased the frequency of Th1 and Th17 cells, particularly in the two-dose treatment group of rats at 8 weeks post-induction (Fig. 3F–I, Additional file 1: Fig. S1C–F). Immunohistochemistry revealed that hUCMSC treatment reduced CD3 + T cell infiltrates in heart tissues of rats (Additional file 2: Fig. S2). Interestingly, treatment with two-dose IDO-OE hUCMSCs greatly enhanced the therapeutic effects on modulating cardiac T cell infiltrates, which were dramatically mitigated by treatment with IDO-KD hUCMSCs. Similarly, treatment with single-dose hUCMSCs increased IL-10, IL-5, and IL-33 mRNA transcripts in the heart of rats, relative to that of the DCM group of rats (Fig. 3J). Treatment with two-dose hUCMSCs also significantly increased the relative levels of IL-10 and TGF-β, but not IL-4, IL-5, IL-13, and IL-33 mRNA transcripts in the heart of rats, which were modulated by IDO-OE or IDO-KD, compared with the single-dose group. Moreover, compared with the NC group, the DCM group of rats exhibited significantly higher levels of cardiac IFN-γ, IL-12Rβ2, IL-2, TNF-α, IL-17, and CCR5 mRNA transcripts, which were significantly mitigated or abrogated by treatment with single- or two-dose hUCMSCs (Fig. 3K). While treatment with two-dose IDO-OE hUCMSCs further decreased the relative levels of those pro-informatory mediator mRNA transcripts, except for the relative levels of IL-17 mRNA transcripts, treatment with IDO-KD hUCMSCs dramatically rescued the levels of those pro-inflammatory mediator mRNA transcripts, except for slightly reduced levels of cardiac IFN-γ and TNF-α mRNA transcripts in rats. Finally, Western blot analysis revealed that the relative levels of cardiac IDO protein expression gradually increased in the listed groups, but decreased in the rats received two-dose IDO-KD hUCMSCs (Fig. 3L, M). Collectively, these data indicated that treatment with hUCMSCs inhibited pro-inflammatory Th1 and Th17 responses and enhanced anti-inflammatory Th2 and Treg responses in a trend of dose-dependence and its therapeutic effects were dependent on IDO expression in hUCMSCs.

### Treatment with IDO-OE hUCMSCs modulates the frequency of splenic and mediastinal LN CD4 + T cells in DCM rats

**Fig. 4:**
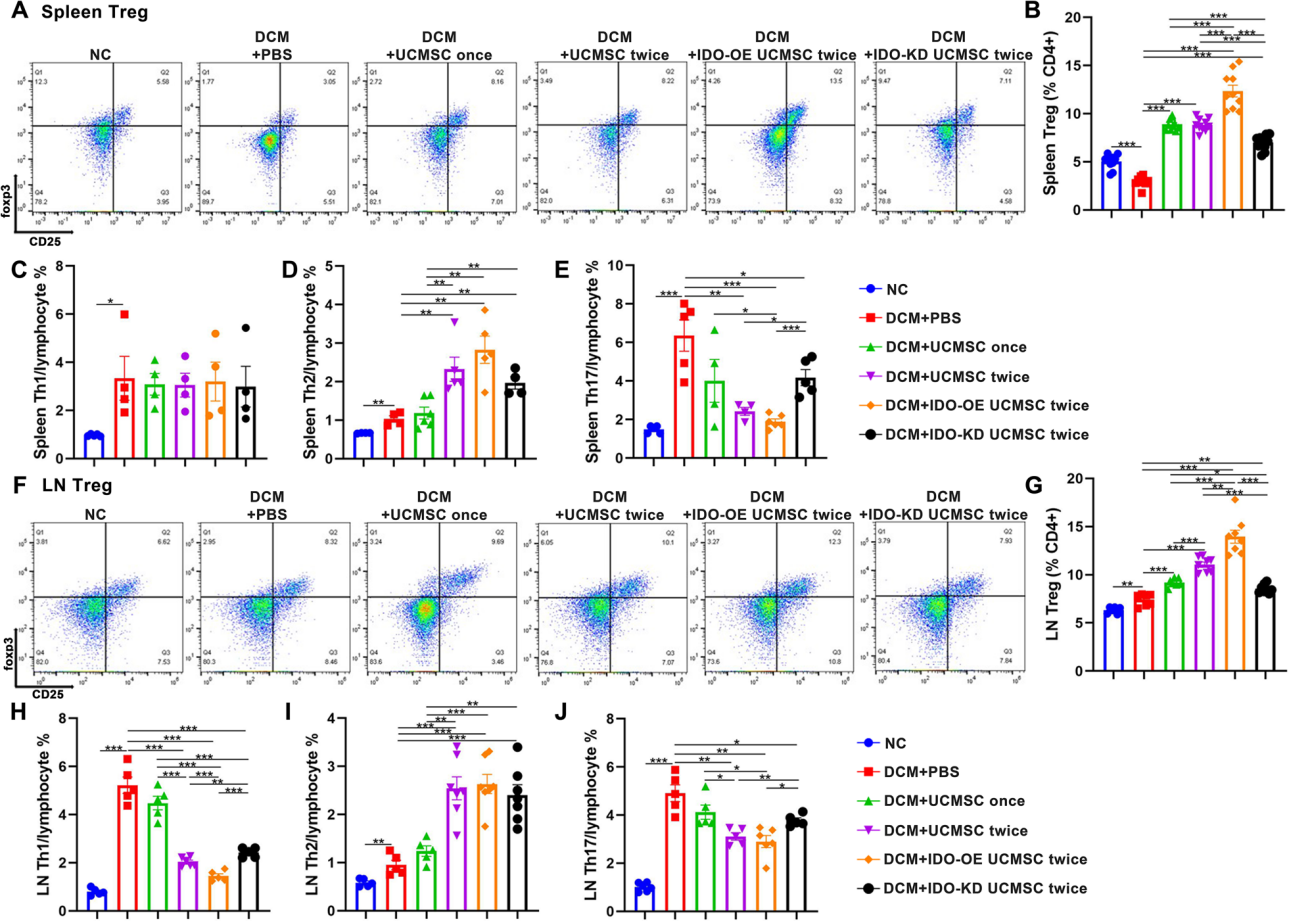
Treatment with IDO-OE hUCMSCs alters the frequency of different subsets of CD4 + T cells in the spleen and mediastinal LNs of DCM rats. **A**, **B** Representative flow cytometry dot plots and quantitative analysis of Tregs in the spleens. **C**–**E** Quantitative analysis of Th1, Th2, and Th17 proportions in the spleens. **F**, **G** Representative flow cytometry dot plots and quantitative analysis of Tregs in the lymph nodes. **H**–**J** Quantitative analysis of Th1, Th2, and Th17 proportions in the lymph nodes. Data are mean ± SEM of each group (*N* = 3–5 rats per group) from at least three separate experiments. *P < 0.05, **P < 0.001, ***P < 0.001 by one-way ANOVA

We further analyzed the frequency of splenic and mediastinal LN Th1, Th2, Th17 cells, and Tregs in rats at 8 weeks post-induction by flow cytometry. As shown in Fig. 4A, B, compared with the NC group, significantly reduced frequency of splenic Tregs was detected in the DCM group of rats and the frequency of splenic Tregs significantly increased in all of the hUCMSC-treated groups. While treatment with IDO-OE hUCMSCs further significantly increased the percentages of splenic Tregs, treatment with IDO-KD hUCMSCs decreased the frequency of splenic Tregs, relative to that of treatment with unmanipulated hUCMSCs in rats. Furthermore, while compared with the NC group, significantly higher frequency of splenic Th1, Th2, and Th17 cells in the DCM group of rats further increased, percentages of splenic Th2 cells were detected in the two-dose hUCMSC-treated groups, particularly in the IDO-OE hUCMSC-treated rats (Fig. 4C–E). Compared with the DCM group, treatment with hUCMSCs failed to later the frequency of splenic Th1 cells. Treatment with two-dose hUCMSCs significantly decreased the percentages of splenic Th17 cells in DCM rats, particularly in the group received IDO-OE hUCMSCs, which were partially rescued in the rats received IDO-KD hUCMSCs. In contrast, the frequency of Tregs in the mediastinal LNs of DCM rats was significantly higher than that in the NC group, while treatment with hUCMSCs further increased the frequency of LN Tregs in rats in a trend of dose-dependence (Fig. 4F, G). Treatment with IDO-OE hUCMSCs further significantly increased the percentages of LN Tregs in rats, while treatment with IDO-KD hUCMSCs dramatically reduced the frequency of LN Tregs in rats, relative to that in other hUCMSCs-treated groups. Moreover, compared with the NC group, the DCM group of rats displayed significantly higher frequency of LN Th1 and Th17 cells (Fig. 4H–J). Treatment with two-dose, but not single-dose, hUCMSCs significantly decreased the frequency of LN Th1 and Th17 cells in rats, and treatment with IDO-OE hUCMSCs further reduced the frequency of LN Th1 and Th17 cells. In contrast, treatment with IDO-KD hUCMSCs partially rescued the percentages of LN Th1 and Th17 cells in rats, relative to that in the rats receiving two-dose hUCMSCs. Furthermore, compared with DCM group, treatment with two-dose hUCMSCs significantly increased the frequency of LN Th2 cells regardless of the levels of IDO expression (Fig. 4I). Similar patterns of the relative levels of splenic and LN Tbet (Th1 cell transcription factor), GATA-3 (Th2 cell transcription factor), RORγt (Th17 cell transcription factor), and FoxP3 (Treg cell transcription factor) gene mRNA transcripts were detected by RT-qPCR in the different groups of rats (Additional file 3: Fig. S3, Additional file 4: Fig. S4). Together, the results indicated that treatment with hUCMSCs, particularly with IDO-OE cells, modulated the frequency of systemic CD4^+^ T cell subsets by inhibiting pro-inflammatory Th1 and Th17 responses, but enhancing anti-inflammatory Th2 and Treg responses in DCM rats.

### Infusion with IDO-OE hUCMSC changes circulating CD4 + T cell subsets and cytokine levels in DCM rats

**Fig. 5:**
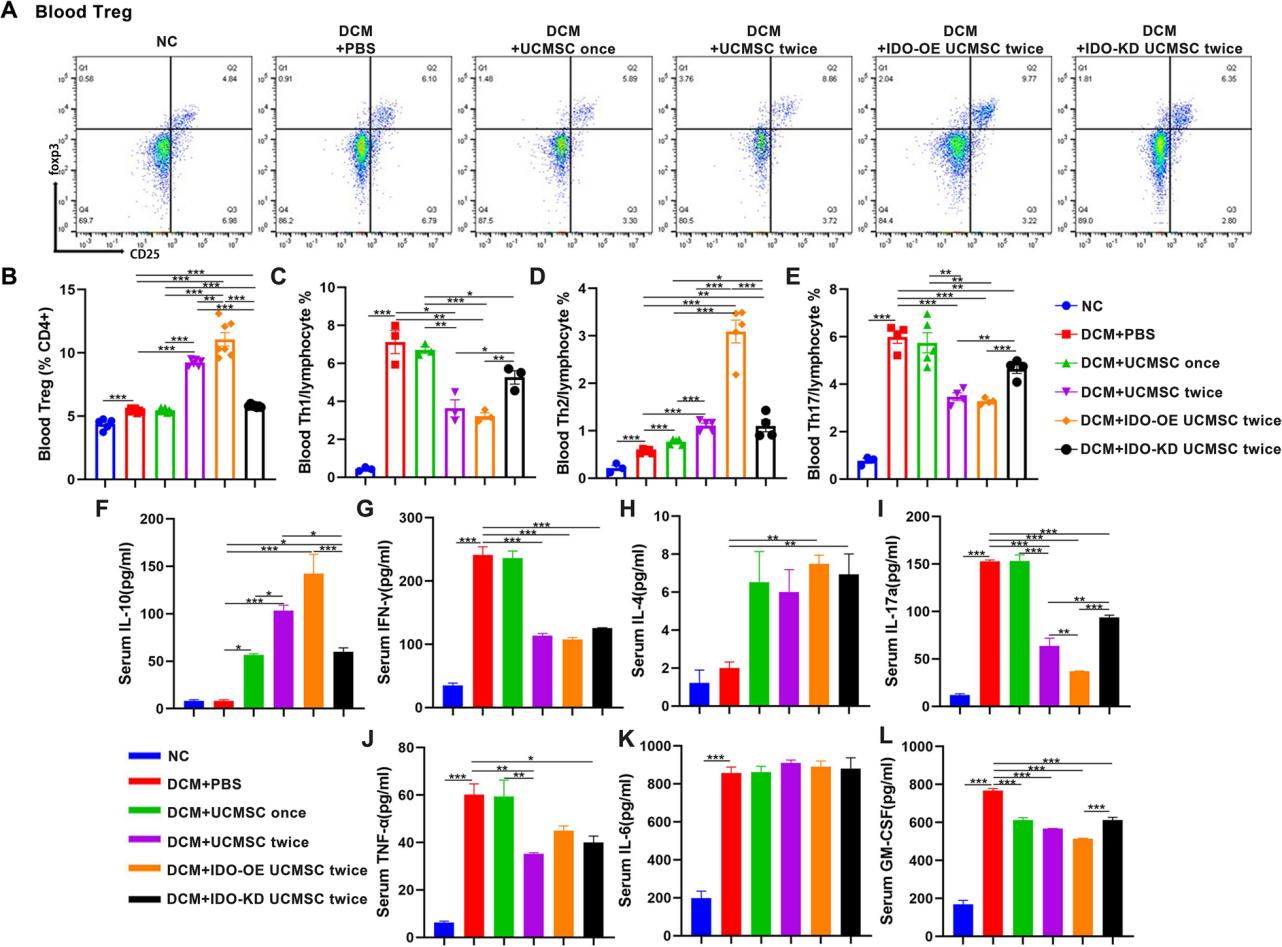
Treatment with IDO-OE hUCMSCs changes the frequency of different subsets of circulating CD4 + T cells in DCM rats. **A**, **B** Representative flow cytometry dot plots and quantitative analysis of peripheral blood Tregs in DCM rats at week 8 post-induction. **C**–**E** Quantitative analyses of Th1, Th2, and Th17 proportions in peripheral blood from DCM rats at week 8 post-induction. **F**–**L** The levels of serum IL-10, IFN-γ, IL-4, IL-17a, TNF-α, IL-6, and GM-CSF in individual rats at 8 weeks post-induction. Data are mean ± SEM of each group (*N* = 3–5 rats per group) from at least two independent experiments. *P < 0.05, **P < 0.001, ***P < 0.001 by one-way ANOVA

First, we longitudinally measured the frequency of circulating Th1, Th2, Th17 cells, and Tregs in the different groups of rats by flow cytometry. As shown in Additional file 5: Fig. S5D–G, following induction of DCM, the frequency of circulating Th1, Th2, Th17 cells, and Tregs gradually increased and the percentages of Tregs peaked at 4 weeks post-induction and declined gradually with time, except that the frequency of Tregs continually increased in the rats receiving two-dose hUCMSCs. Similarly, the frequency of circulating Th2 cells gradually increased in the rats receiving IDO-OE hUCMSCs, but only slightly increased in other groups of DCM rats. In contrast, the percentages of circulating Th1 and Th17 cells gradually increased in the different groups of DCM rats and ranked from the highest in the DCM, single-dose, IDO-KD to the two-dose and IDO-OE groups. Flow cytometry revealed that compared with the NC group, the DCM group of rats at 8 weeks post-induction had significantly higher frequency of circulating Tregs, Th1, Th2, and Th17 cells (Fig. 5A–E, Additional file 5: Fig. S5A–C). The percentages of Tregs significantly increased in the rats with two-dose hUCMSCs and further elevated in the rats receiving IDO-OE hUCMSCs, but reduced in the rats with IDO-KD hUCMSCs (Fig. 5B). Similarly, treatment with hUCMSCs also increased the frequency of Th2 cells in a trend of dose-dependence, particularly in the rats receiving IDO-OE hUCMSCs, but decreased in the rats with IDO-KD hUCMSCs (Fig. 5D). Moreover, treatment with two-dose hUCMSCs significantly decreased the percentages of Th1 and Th17 cells, particularly with IDO-OE hUCMSCs, while treatment with IDO-KD hUCMSCs partially rescued the frequency of Th1 and Th17 cells in rats (Fig. 5C, E). Analysis of serum cytokines indicated that compared with the NC group, the DCM group of rats exhibited significantly higher levels of serum IFN-γ, IL-17, TNFα, IL-6, GM-CFS, but not IL-10 and IL-4 (Fig. 5F–L). Compared with that in the DCM group, treatment with hUCMSCs increased the levels of serum IL-10, particularly with IDO-OE hUCMSCs, while treatment with IDO-KD hUCMSCs decreased serum IL-10 levels in rats, relative to that in the rats with two-dose hUCMSCs (Fig. 5F). Treatment with two-dose hUCMSCs significantly decreased the levels of serum IFN-γ, IL-17, and TNF-α, particularly with hUCMSCs, while treatment with IDO-KD hUCMSCs partially rescued serum IL-17 and GM-CSF, but not IFN-γ and TNF-α, levels in rats, relative to that in the DCM group (Fig. 5G, I, J, L). Treatment with IDO-OE or IDO-KD hUCMSCs significantly increased serum IL-4 although treatment with any type of hUCMSCs failed to alter the levels of serum IL-6 in rats (Fig. 5H–K). Thus, treatment with IDO-OE hUCMSCs significantly enhanced circulating anti-inflammatory Th2 and Treg responses, but reduced pro-inflammatory Th1 and Th17 responses in rats.

### Treatment with IDO-OE hUCMSCs mitigates the severity of myocardial fibrosis in DCM rats

**Fig. 6:**
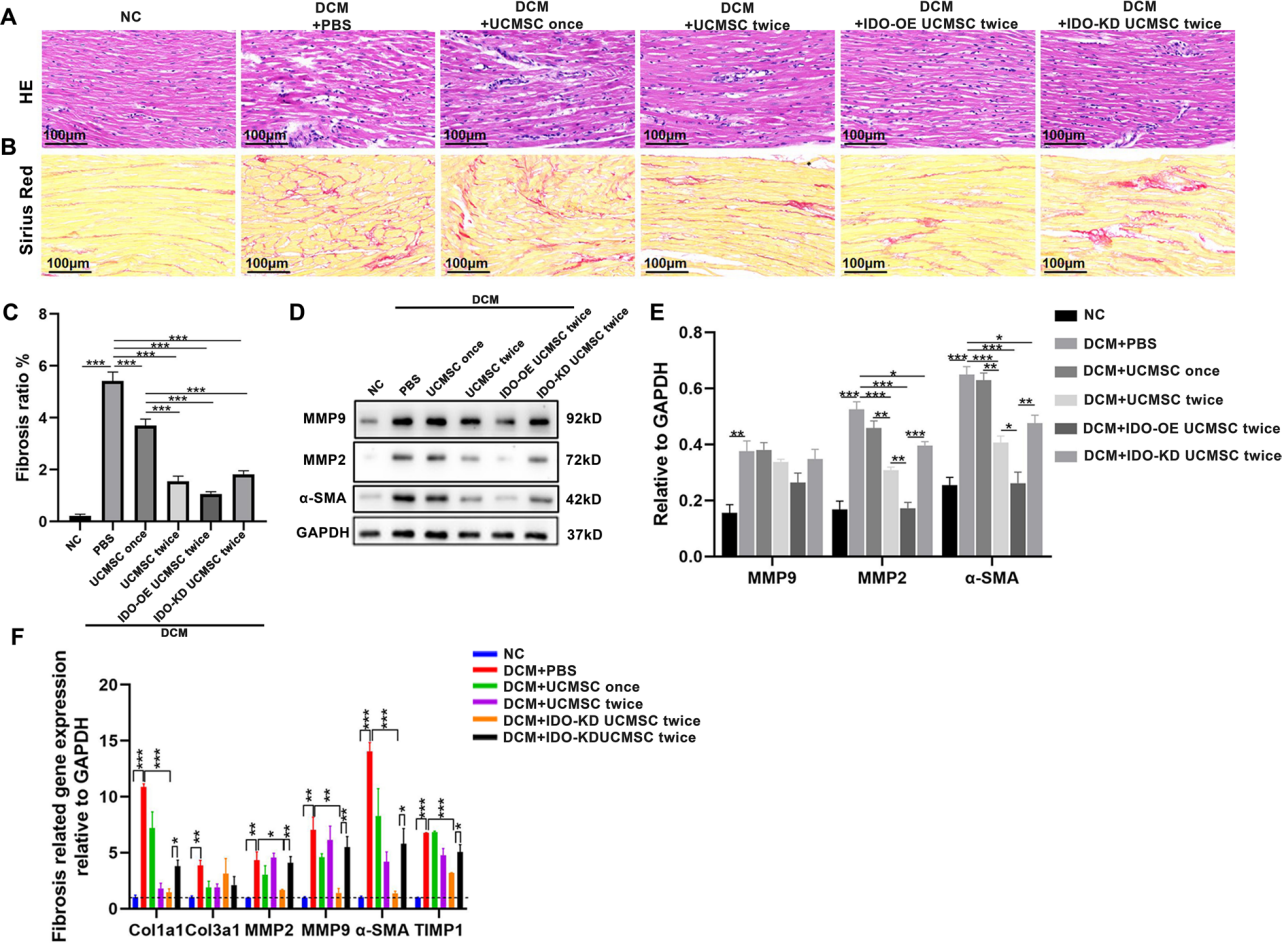
IDO-OE hUCMSC administration reduces the degrees of myocardial fibrosis in DCM rats. **A** Hematoxylin and eosin (H&E) staining of morphological changes in rat heart samples, scale bar = 100 μm. **B** Sirius Red staining of collagen fibers in rat heart specimens, scale bar = 100 μm. **C** Quantitative analysis of fibrosis severity, based on Sirius Red staining. **D**, **E** Western blot analysis of MMP2, MMP9, and α-SMA expression in heart tissues of rats. **F** RT-qPCR analyses of the relative mRNA transcripts of genes related to fibrosis. Data are mean ± SEM of each group (*N* = 3–5 rats per group) from at least two independent experiments. *P < 0.05, **P < 0.001, ***P < 0.001 by one-way ANOVA

Because long-term DOX exposure can induce myocardial fibrosis, we tested whether treatment with hUCMSCs could modulate the severity of myocardial fibrosis in rats. H&E staining displayed obvious cardiomyocyte degeneration and injury, which were reduced in the rats receiving hUCMSCs, particularly in those with IDO-OE hUCMSCs (Fig. 6A). Sirius staining exhibited that compared with the NC group, many collagen fibers were presented in cardiac tissues of the DCM group of rats. While the collagen deposition was significantly reduced in the rats receiving hUCMSCs, particularly in those receiving IDO-OE hUCMSCs (Fig. 6B–C). However, the anti-fibrotic effect of hUCMSCs was partially mitigated by treatment with IDO-KD hUCMSCs. Western blot analysis revealed that while the relative levels of MMP9, MMP2, and α-SMA expression in heart tissues of the DCM group of rats were significantly higher than that in the NC group, the levels of MMP2 and α-SMA, but not MMP9, were significantly reduced in the rats receiving two-dose hUCMSCs, particularly in those with IDO-OE hUCMSCs (Fig. 6D–E). Treatment with IDO-KD hUCMSCs only partially rescued the levels of MMP2 and α-SMA expression in heart tissues of DCM rats. RT-qPCR revealed that compared with the NC group, significantly higher levels of *Col1a, Col3a, MM2, MMP9, α-SMA*, and *TIMP1* mRNA transcripts were detected in heart tissues of DCM group, which were significantly reduced, except for *Col3a*, in the rats with IDO-OE hUCMSCs (Fig. 6F). However, treatment with IDO-KD hUCMSCs significantly rescued the relative levels of *Col1a, MM2, MMP9, α-SMA*, and *TIMP1* mRNA transcripts in heart tissues of DCM rats. Therefore, treatment with IDO-OE hUCMSCs significantly mitigated the DCM-related myocardial fibrosis in DCM rats.

### Treatment with IDO-OE hUCMSCs reduces cardiomyocyte apoptosis in DCM rats

**Fig. 7:**
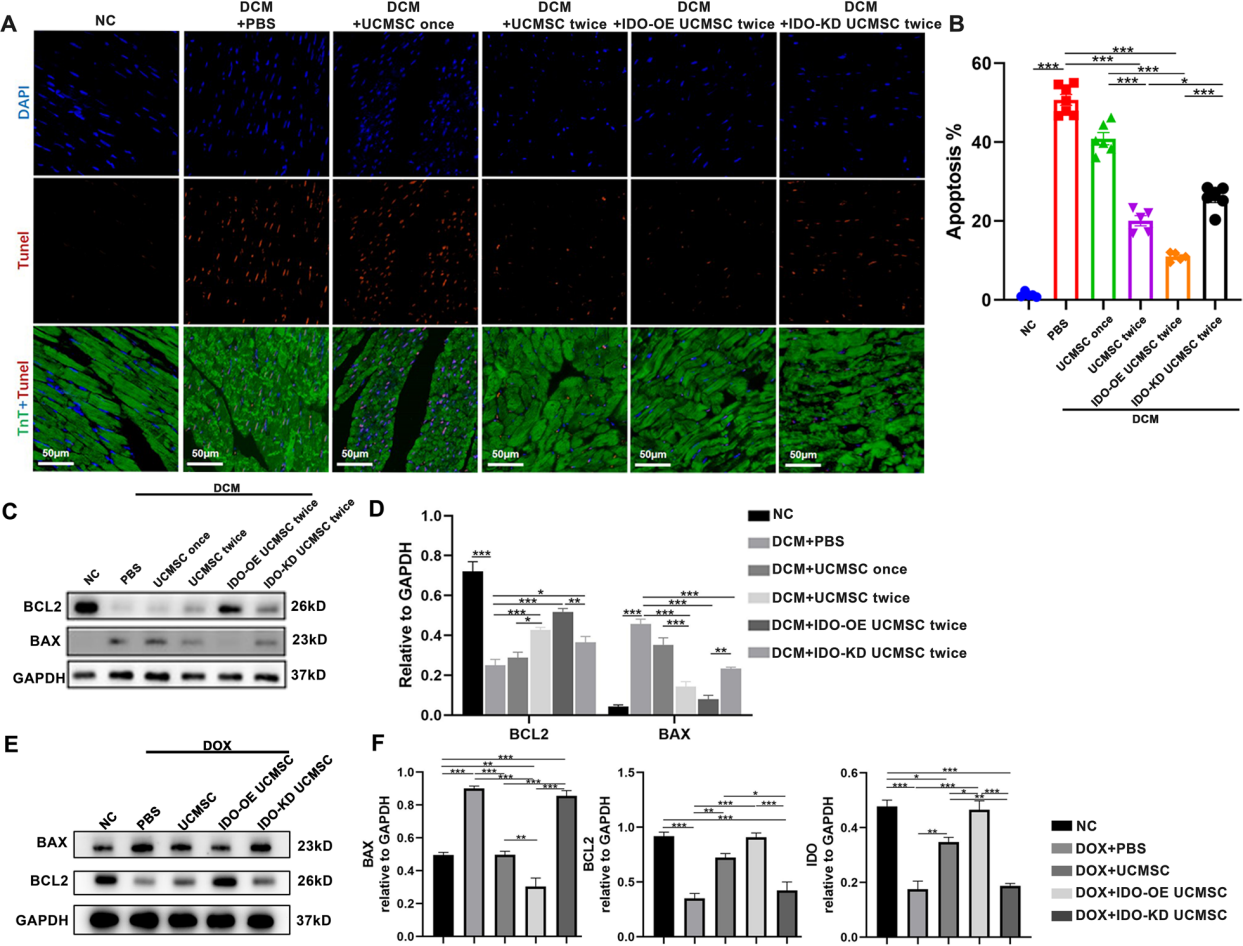
Treatment with IDO-OE hUCMSC inhibits cardiomyocyte apoptosis in DCM rats. **A**, **B** TUNEL (red) and anti-TnT (green) co-staining of heart samples and quantitative analysis of the percentages of apoptotic cardiomyocytes in rats at 8 weeks post-induction, scale bar = 50 μm. **C**, **D** Western blot and quantitative analysis of the relative levels of BAX and BcL-2 expression in heart tissues of rats. **E**, **F** Western blot analysis of the relative levels of IDO, BAX and BcL-2 expression in cardiomyocytes. Data are mean ± SEM of each group (*N* = 3–5 rats per group) from two separate experiments. *P < 0.05, **P < 0.001, ***P < 0.001 by one-way ANOVA

Finally, we examined the effect of treatment with hUCMSCs on cardiomyocyte apoptosis in the different groups of rats. TUNEL assays indicated that while there were few apoptotic TnT + TUNEL + cardiomyocytes in the NC group, many apoptotic cardiomyocytes were detected in the DCM group of rats (Fig. 7A, B). Treatment with two-dose hUCMSCs significantly decreased the percentage of apoptotic cardiomyocytes in DCM rats, particularly with IDO-KD hUCMSCs, but treatment with IDO-KD hUCMSCs partially rescued the frequency of apoptotic cardiomyocytes in DCM rats. Western blot revealed that compared with the NC group, the DCM group of rats displayed higher levels of Bax, but lower levels of BcL-2 expression in their hearts (Fig. 7C, D). Treatment with hUCMSCs, particularly with IDO-OE hUCMSCs, significantly decreased the relative levels of Bax expression, but increased BcL-2 in heart tissues of DCM rats. Treatment with IDO-KD hUCMSCs failed to significantly alter the relative levels of Bax, and BcL-2 expression in heart tissues of DCM rats, relative to that in the DCM group of rats. Similarly, co-culturing with hUCMSCs, particularly with IDO-OE hUCMSCs, significantly mitigated the DOX-induced cardiomyocyte apoptosis by decreasing pro-apoptotic Bax expression, but enhancing anti-apoptotic BcL-2 expression in vitro (Fig. 7E, F). These data clearly demonstrated that treatment with IDO-OE hUCMSCs significantly minimized cardiomyocyte apoptosis in DCM rats.

## Discussion

**Fig. 8:**
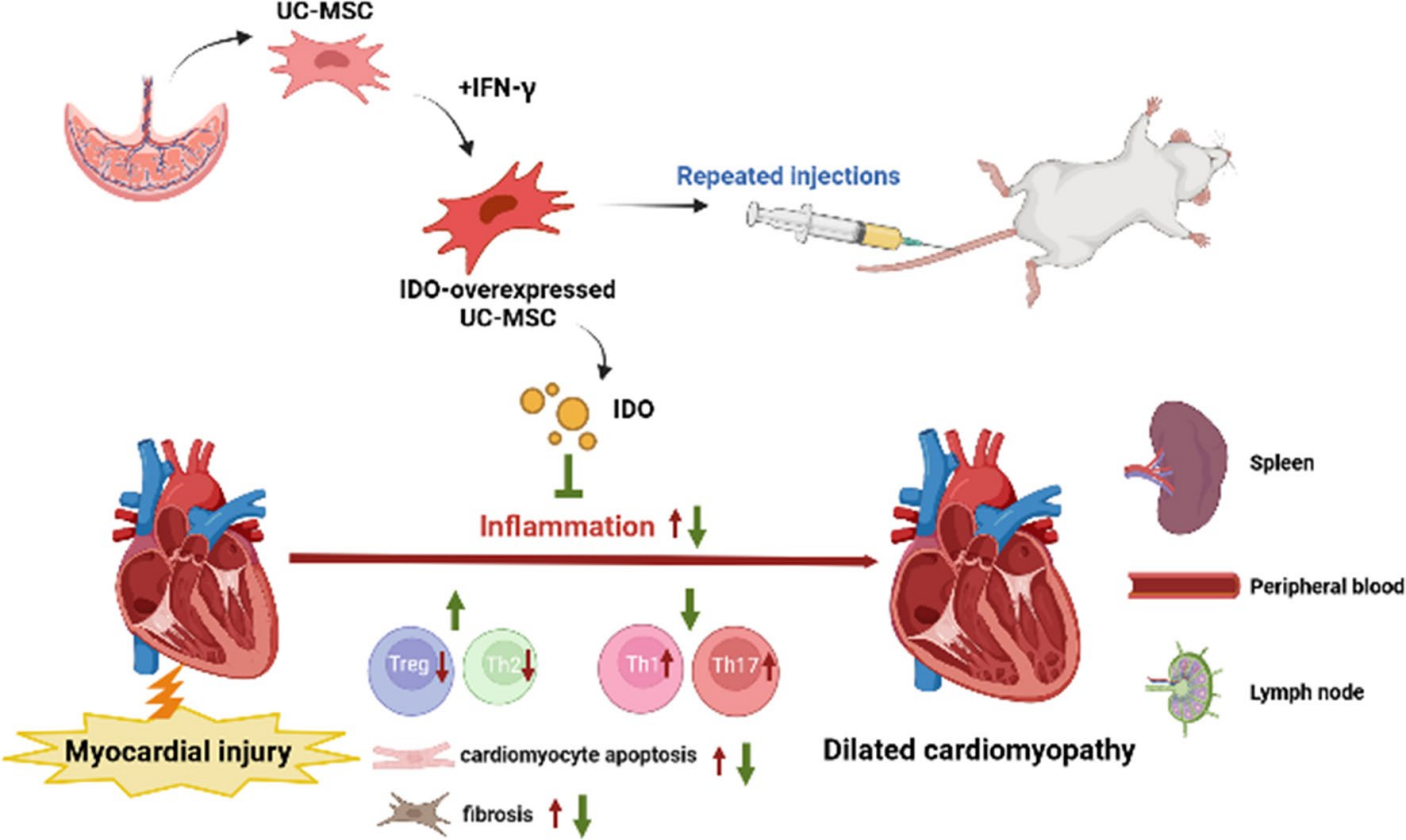
Central illustration. Two doses of human umbilical cord mesenchymal stem cells alleviate cardiac dysfunction and inflammation in the dilated cardiomyopathy rat model by upregulating indoleamine 2,3-dioxygenase expression. Doxorubicin exposure led to cardiac dysfunction in the rat dilated cardiomyopathy model along with systemic and persistent low-grade inflammation in the heart, peripheral blood, lymph nodes, and spleen. Intravenous treatment with two doses of hUCMSCs alleviated cardiac dysfunction and modulated the imbalance of anti-inflammatory Treg and Th2 and pro-inflammatory Th1 and Th7 responses. Furthermore, IDO-OE hUCMSC treatment exerted more pronounced effects compared with the unmanipulated hUCMSC group in reducing cardiomyocyte apoptosis, and fibrosis, while treatment with IDO-KD hUCMSCs reduced their therapeutic effects. Therefore, we hypothesize that IDO and its metabolites may be important for hUCMSCs to regulate the immune-inflammatory responses in the DOX-induced heart failure

In this study, the main finding indicated that (1) treatment with two-dose hUCMSCs achieved a better therapeutic efficacy than single-dose therapy in restoring cardiac functions, attenuating cardiac dilation, and reducing cardiomyocyte apoptosis and fibrosis; (2) two-dose therapy with hUCMSCs had stronger capacity than single-dose therapy to significantly increase the frequency of Tregs in the heart and peripheral blood and cardiac expression of IDO; (3) hUCMSC treatment significantly decreased the number of Th1 cell infiltrates in the heart, dependent on IDO expression levels in hUCMSCs; and (4) induction of IDO over-expression in hUCMSCs enhanced their suppressive activity by enhancing anti-inflammatory Treg and Th2 responses and inhibiting pro-inflammatory Th1 and Th17 responses in DCM rats. Hence, during the pathogenic process of DCM, the damaged cardiomyocytes induce inflammation, which recruits pro-inflammatory Th1, Th17, and other cell infiltrates that secrete pro-inflammatory IFN-γ and TNF-α as well as others to upregulate IDO expression in the lesion, including infiltrates and treated hUCMSCs. The increased IDO expression and its mediated metabolites enhance Treg differentiation to enhance Treg and Th2 responses, inhibiting pro-inflammatory T cell responses, limiting cardiac dilation and restoring LV function (Central illustration: Fig. 8). Therefore, our findings may shed lights on the pathogenesis of DCM and uncover new therapeutic targets for intervention of DCM.

DCM is the most common cardiomyopathy worldwide with structural and functional abnormalities [29]. Currently, there are few effective therapeutic strategies for DCM. HUCMSCs have the potential for multipotent differentiation and have been considered as a promising cell-based therapy [30]. A previous study has shown that treatment with single-dose hUCMSCs can improve LV function, functional status, and life quality of patients with HF with reduced ejection fraction (HFrEF) [31]. However, this therapeutic strategy does not significantly reduce mortality and hospital admission of HF patients. In DCM animal models, treatment with single-dose hUCMSCs preserves cardiac function [6, 7, 9, 10]. A single treatment with MSCs may be modestly effective due to its short-lived nature and potentially obstructed in the lung, making the cells hard to reach the targeted lesion [12, 32]. Hence, repeated administration of MSCs may be necessary and quite effective [12, 33]. Actually, repeated infusions with BM-MSCs improve cardiac function in DOX-induced DCM rats [8]. However, it is still unclear whether therapeutic efficacy of repeated infusions with hUCMSCs is better than single-dose therapy. In this study, we found that two-dose infusion of hUCMSCs resulted in better therapeutic efficacy than single-dose therapy in inhibiting inflammation, enhancing anti-inflammatory Treg and Th2 responses, restoring LV function, and limiting myocardial dilation in rats. Our novel findings fill the knowledge gap and may help in designing new MSC-based therapeutic strategies for DCM and other types of inflammatory diseases.

Inflammation is crucial for the development and progression of HFrEF, which is associated with elevated levels of circulating pro-inflammatory cytokines [4]. MSC administration attenuates the process of inflammatory cardiomyopathy in rodents [5, 34]. Several studies have shown that single-dose hUCMSC therapy improves cardiac function and structure in animals with HF [35–37], associated with reducing cardiomyocyte apoptosis, myocardial fibrosis, and inflammation by modulating cytokine responses. We found that two-dose infusion of hUCMSCs significantly increased anti-inflammatory Treg and Th2 responses and inhibited pro-inflammatory Th1 and Th17 responses in DCM rats, accompanied by reducing cardiac fibrosis and cardiomyocyte apoptosis. These may be directly attributed to inhibitory cytokines secreted by infused hUCMSCs and/or indirectly mediated by the hUCMSCs-enhanced Treg responses. Therefore, infusion of two-dose hUCMSCs led to potent anti-inflammatory, anti-apoptotic, and anti-fibrotic effects in DCM rats.

IDO and its mediated metabolites can induce the differentiation of Tregs, which suppress pro-inflammatory T cell responses [15]. A previous study has indicated that IDO and its metabolite of KYNA are responsible for the TSG-6-mediated anti-inflammatory effects of human MSCs in a mouse model of acute lung injury induced by lipopolysaccharide [13]. In this study, we observed that treatment with two-dose hUCMSCs significantly upregulated IDO expression and Treg infiltrates in the heart of DCM rats. Furthermore, treatment with IDO-OE hUCMSCs further enhanced anti-inflammatory activities and restored cardiac function by increasing Treg responses, while treatment with IDO-KD hUCMSCs had a reduced therapeutic effect on cardiac function in DCM rats. The powerful therapeutic effects of IDO-OE hUCMSCs may stem from the fact that IDO and its metabolites can induce strong Treg responses that attenuate pro-inflammatory responses to restore cardiac function. These findings support the notion that IDO is critical for the control of aberrant inflammation and may be a therapeutic target for intervention of inflammatory diseases.

Infusion of MSCs is safe for humans and obviates the invasive delivery of cells to the target tissues, making cell therapy simpler, and also feasible for multiple dosing [12]. Our findings indicated that infusion of two-dose hUCMSCs effectively reduced inflammation in the heart to restore cardiac function in DCM rats. Therefore, our findings may help in designing a multiple dosing delivery of cell therapy in future preclinical and clinical trials.

## Conclusion

Our data indicated that treatment with two-dose hUCMSCs, particularly with IDO-OE hUCMSCs, was more effective than single-dose therapy for restoring LV function in DCM rats, which were associated with enhancing anti-inflammatory Treg responses and inhibiting pro-inflammatory Th1 and Th17 responses. The therapeutic efficacy of hUCMSCs was dependent on their IDO expression levels. Therefore, our findings may be valuable for design of clinical trials to test the therapeutic efficacy of hUCMSCs in HF and other inflammatory diseases.

## Supplementary Information

**Additional file 1: Figure S1**. (A) A line graph of EF% in six groups of rats measured by echocardiography at 0, 2, 4, 6, or 8 weeks post induction. (B) A line graph of E/E′ in six groups of rats measured by echocardiography at 0, 2, 4, 6 or 8 weeks post induction. (C–F) Representative flow-cytograms of the frequency of Treg, Th1, Th2, and Th17 cells in the heart of rats at week 8 post induction. Data are mean ± SEM of each group (*N* = 3–5 rats per group).

**Additional file 2: Figure S2**. (A, B) Immunohistochemical and quantitative analysis of CD3 expression in rat hearts at 8 weeks post induction. (C) Immunohistochemical analysis of CD68 expression in rat hearts at 8 weeks post induction. Data are mean ± SEM of each group (*N* = 3–5 rats per group) from at least two independent experiments. *P < 0.05, **P < 0.001, ***P < 0.001 by one-way ANOVA.

**Additional file 3: Figure S3**. (A–C) Representative flow cytometry charts of the frequency of Th2, Th1, and Th17 cells in the spleens of rats at 8 weeks post induction. (D-G) RT-qPCR analyses of the relative levels of FOXP3 (Treg), GATA3 (Th2), TBET (Th1), and RORγΓ (Th17) mRNA transcripts in the spleens of rats at 8 weeks post induction. Data are mean ± SEM of each group (*N* = 3–5 rats per group). *P < 0.05, **P < 0.001, ***P < 0.001 by one-way ANOVA.

**Additional file 4: Figure S4**. (A–C) Representative flow cytometry charts of the frequency of Th2, Th1, and Th17 cells in the lymph nodes of rats at 8 weeks post induction. (D-G) RT-qPCR analyses of the relative levels of FOXP3 (Treg), GATA3 (Th2), TBET (Th1), and RORγΓ (Th17) mRNA transcripts in the lymph nodes of rats at 8 weeks post induction. Data are mean ± SEM of each group (*N* = 3–5 rats per group). *P < 0.05, **P < 0.001, ***P < 0.001 by one-way ANOVA.

**Additional file 5: Figure S5**. (A–C) Representative flow cytometry charts of the frequency of Th2, Th1, and Th17 cells in peripheral blood of rats at 8 weeks post induction. (D–G) Line graphs of the proportions of peripheral blood Treg, Th2, Th1 and Th17 cells measured by flow cytometry in rats at 0, 2, 4, 6 or 8 weeks post induction. Data are mean ± SEM of each group (*N* = 3–5 rats per group).

## Data Availability

The datasets analyzed in the current study are available upon reasonable request from the corresponding authors.
