# Supplementary figures and images for: Infusion of two-dose mesenchymal stem cells is more effective than a single dose in a dilated cardiomyopathy rat model by upregulating indoleamine 2,3-dioxygenase expression
Source: Stem Cell Res Ther. 2022 Aug 12;13:409. doi: 10.1186/s13287-022-03101-w (PMC9373305; doi:10.1186/s13287-022-03101-w)

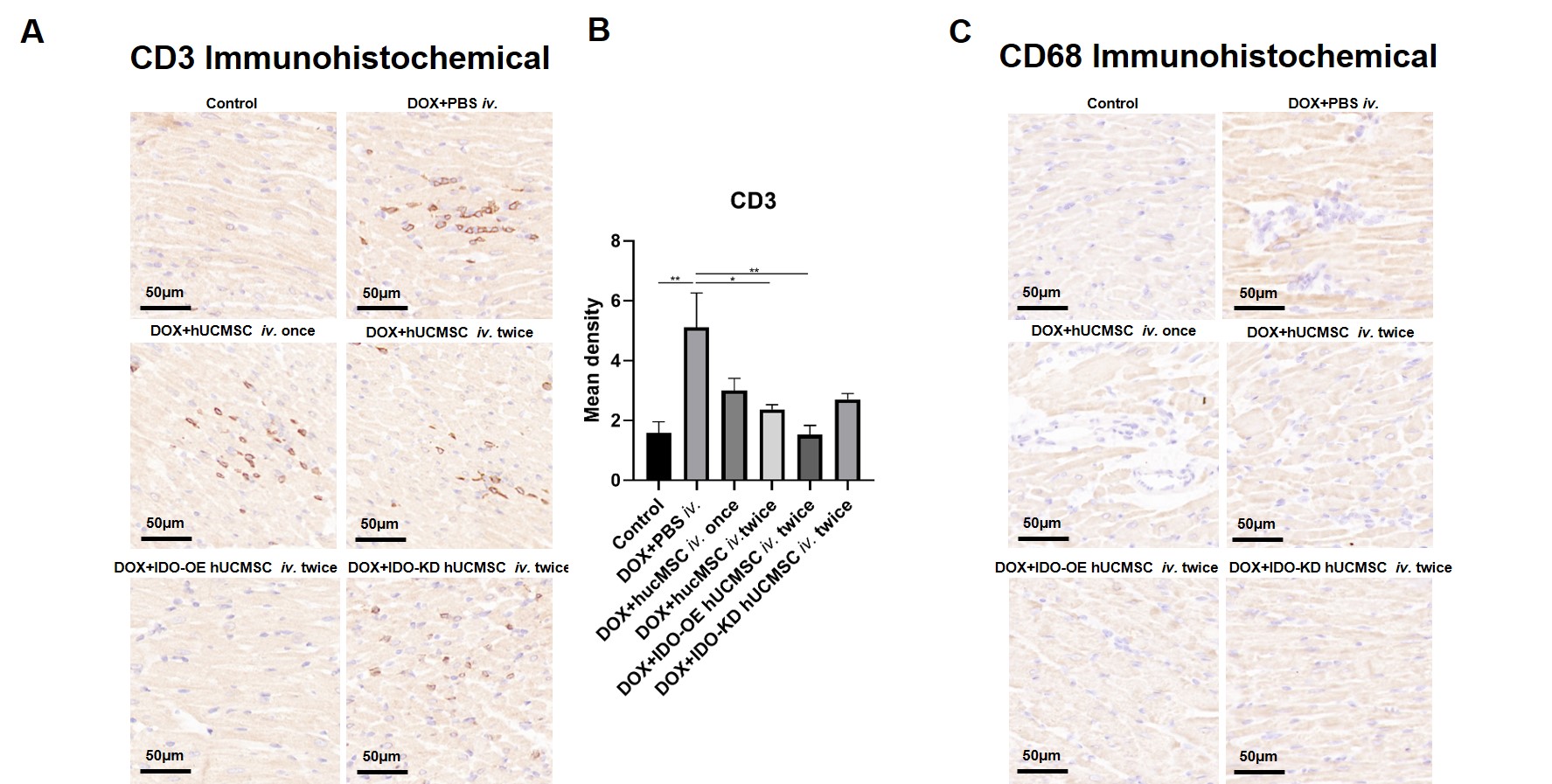

Supplement: Supplementary file 2 — Additional file 2: Figure S2. (A, B) Immunohistochemical and quantitative analysis of CD3 expression in rat hearts at 8 weeks post induction. (C) Immunohistochemical analysis of CD68 expression in rat hearts at 8 weeks post induction. Data are mean ± SEM of each group (N = 3–5 rats per group) from at least two independent experiments. *P < 0.05, **P < 0.001, ***P < 0.001 by one-way ANOVA. [file 13287_2022_3101_MOESM2_ESM.jpg]
